# Hypertension and cardiovascular risk factor management in a multi-ethnic cohort of adults with CKD: a cross sectional study in general practice

**DOI:** 10.1007/s40620-021-01149-0

**Published:** 2021-11-16

**Authors:** Edianne Monique Carpio, Mark Ashworth, Elham Asgari, Catriona Shaw, Patricia Schartau, Stevo Durbaba, Dorothea Nitsch, Mariam Molokhia

**Affiliations:** 1 Department of Population Health Sciences, King’s College London, London, England; 2 Renal Department, Guy’s and St Thomas’ NHS Foundation Trust, London, England; 3 Renal Department, King’s College Hospital, London, England; 4 Department of Primary Care & Population Health, Institute of Epidemiology & Health, University College London, London, England; 5 Department of Non-Communicable Disease Epidemiology, London School of Hygiene & Tropical Medicine, London, England

**Keywords:** CKD, Hypertension, Ethnicity, CVD, Undiagnosed, African

## Abstract

**Background:**

Hypertension, especially if poorly controlled, is a key determinant of chronic kidney disease (CKD) development and progression to end stage renal disease (ESRD).

**Aim:**

To assess hypertension and risk factor management, and determinants of systolic blood pressure control in individuals with CKD and hypertension.

**Design and setting:**

Cross-sectional survey using primary care electronic health records from 47/49 general practice clinics in South London.

**Methods:**

Known effective interventions, management of hypertension and cardiovascular disease (CVD) risk in patients with CKD Stages 3–5 were investigated. Multivariable logistic regression analysis examined the association of demographic factors, comorbidities, deprivation, and CKD coding, with systolic blood pressure control status as outcome. Individuals with diabetes were excluded.

**Results:**

Adults with CKD Stages 3–5 and hypertension represented 4131/286,162 (1.4%) of the total population; 1984 (48%) of these individuals had undiagnosed CKD without a recorded CKD clinical code. Hypertension was undiagnosed in 25% of the total Lambeth population, and in patients with CKD without diagnosed hypertension, 23.0% had systolic blood pressure > 140 mmHg compared with 39.8% hypertensives, p < 0.001. Multivariable logistic regression revealed that factors associated with improved systolic blood pressure control in CKD included diastolic blood pressure control, serious mental illness, history of cardiovascular co-morbidities, CKD diagnostic coding, and age < 60 years. African ethnicity and obesity were associated with poorer systolic blood pressure control.

**Conclusion:**

We found both underdiagnosed CKD and underdiagnosed hypertension in patients with CKD. The poor systolic blood pressure control in older age groups ≥ 60 years and in Black African or obese individuals is clinically important as these groups are at increased risk of mortality for cardiovascular diseases.

**Graphic abstract:**

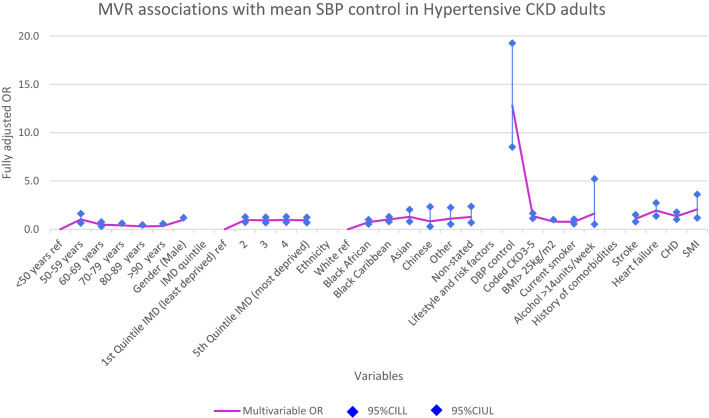

**Supplementary Information:**

The online version contains supplementary material available at 10.1007/s40620-021-01149-0.

## Introduction

### CKD and hypertension

The diagnosis of chronic kidney disease (CKD) is mainly based on estimated glomerular filtration rate (eGFR) < 60 ml/min/1.73m^2^ for at least 3 months, even if the broader definition includes all persistent alterations of renal morphology and of blood or urine composition [1]. Key risk factors for CKD include hypertension, cardiovascular disease (CVD), and diabetes [2], and CKD is associated with significant morbidity and mortality, in particular in these groups [2, 3].

CKD is common in England, an estimated 6.1% people aged 16 ≥ years are living with CKD (defined as eGFR < 60 ml/min/1.73m^2^), and this is projected to include 3.2 million people in 2021 [4]. The treatment of CKD Stage 3–5 poses a considerable financial burden, and the lifetime the lost health-related quality of life is estimated as £7.18 billion in England [5]. This includes the high cost of renal replacement therapy (RRT) for survivors with end-stage renal disease and cardiovascular disease complications. In UK primary care, the Quality and Outcomes Framework (QOF) incentivises maintaining a CKD register with regular renal monitoring, which now includes classification of GFR categories “G3a to G5” (based on eGFR), although recommendations for BP targets < 140/90, and Albumin Creatinine Ratio (ACR) testing have been dropped [6]. However, persistent albuminuria prevalence (elevated urinary ACR with normal eGFR) in CKD stages 1 and 2 is estimated as high as 10% [7] and is associated with a doubling in mortality risk [8].

Data from the national CKD audit (NCKDA) report suggest that individuals with CKD and hypertension may not be well managed [9]. However, the NCKDA (gathering a largely White population) was unable to examine ethnic inequalities. The NCKDA audit suggests that 30% of CKD is undiagnosed (i.e. not formally coded), especially at younger ages, resulting in poorer quality of care [9]. The National Institute for Health and Care Excellence (NICE) Guidelines recommend regular renal function monitoring for individuals with hypertension, diabetes, and cardiovascular disease (CVD). In these groups, CKD is therefore more likely to be detected, as this is supported by QOF incentives for disease monitoring, including renal function.

Hypertension, especially if poorly controlled [10, 11] is a key determinant of CKD development and progression, and is present in the majority of people with diagnosed CKD (89%) in England [12, 13]. Antihypertensive medications, such as angiotensin receptor blockers (ARBs) and angiotensin-converting-enzyme inhibitors (ACEIs), are also currently recommended by the NICE (National Institute for Health and Care Excellence) guidelines as first line management for proteinuric CKD and for non proteinuric CKD in patients with hypertension [14], although recent findings suggest that these medications are under-prescribed [10, 15].

Known markers of CKD progression (measured by eGFR decline) include proteinuria [16–18], and blood pressure control has been shown to reduce proteinuria, and delay eGFR decline [19–21]. Blood pressure control has been reported to be poor in patients with CKD Stage 3 [15], but little is known in later stages of CKD. Recognised risk factors for CKD include age, sex, ethnicity, hypertension, diabetes, cardiovascular disease, smoking, obesity and non-steroidal anti-inflammatory drug (NSAID) use [22, 23].

Patients with CKD and hypertension alone are managed and captured differently under Quality Outcome Framework, compared to those with CKD and diabetes. The NCKDA report has previously published differences in management by diabetes status. To our knowledge, there has been limited focus on the management of CKD in the UK by hypertension status. This study aimed to assess hypertension and risk factor management, and determinants of systolic blood pressure (SBP) control in individuals with CKD and hypertension HT.

## Methods

### Study design

Cross-sectional study of non-diabetic individuals with CKD.

### Data sources

We used a dataset derived from general practice electronic health records (EHRs), Lambeth DataNet (LDN), south London, extracted from patient records from 1990 until October 2013. LDN contains clinical data, prescription data, laboratory data, and demographic information, such as age, sex, and ethnicity, based on categories of the UK 2001 census, CVD risk factors and co-morbidities. We investigated demographic factors, comorbidities, and other interventions considered as potentially effective, in a population identified as having CKD Stage 3–5, with and without hypertension.

### Study population

Individuals with CKD Stage 3–5 (defined based on the criteria reported below). Patients with diabetes were excluded, as they have different profiles of proteinuria and cardiovascular risk [24]. The study was carried out using anonymised data from patients registered in 47/49 general practices, in an ethnically and socially diverse population of 286,162 adults (≥ 18 years), followed-up in Lambeth, south London.

### Identification of CKD

Our CKD Stage 3–5 cohort comprised diagnosed and undiagnosed cases. Diagnosed CKD status was determined using QOF incentive scheme CKD descriptive codes, plus codes for dialysis or renal transplantation (Appendix Table S1). Non-diagnosed CKD was defined, in the absence of the previous codes, based on the latest two readings ≥ 90 days apart of eGFR levels < 60 ml/min/1.73m^2^ calculated from serum creatinine using the modified four-variable Modification of Diet in Renal Disease (MDRD) equation adjusted for sex and, when appropriate, Black African or Black-Caribbean ethnicity [4]. We excluded miscoded patients, who after use of the ethnic correction factor had an eGFR > 60 ml/min/1.73m^2^. Hypertensive CKD is defined as individuals with CKD and hypertension.

### Identification of hypertension

Individuals were defined as hypertensive if they were included In the hypertension Quality Outcome Framework (QOF) register.

### Risk factor management

Risk factor management and CKD prevalence by stage was investigated. Appropriate systolic blood pressure control was defined as below 140/90 based on the mean of the two latest readings, in individuals with CKD, according to NICE guidelines [14]. For patients with urinary albumin creatinine ratio > 70 mg/mmol, BP targets were more stringent, < 130/80 mmHg [25]. As we were interested in modifiable lifestyle risk factor management, current smoking status and BMI were included in the analysis.

### Covariates

We examined the following factors: age, gender, ethnicity, deprivation (Index of Multiple Deprivation 2015), and selected comorbidities likely to influence SBP control including coronary heart disease (CHD), heart failure (HF), stroke and serious mental illness, (SMI). Other measured factors were: diastolic blood pressure (DBP) control, prescribed ACEIs or ARBs, calcium channel blockers (CCBs), statins, NSAIDs and COX-2 inhibitors, (COXIBs) and lifestyle factors smoking and obesity. High alcohol intake was defined as > 14 units/week. BMI was determined according to the World Health Organization (WHO) classification [26]. Ethnicity was self-reported and aggregated into 7 categories: White, African, Black Caribbean, South Asian, Chinese, Other, and Non-stated. Proteinuria measurements were incomplete and therefore were not included.

The primary outcome was systolic blood pressure control; the secondary outcome was CVD risk factor control in patients with CKD.

### Analysis

A cross-sectional study design was used to describe demographic factors, comorbidities, and other interventions considered as being potentially effective (prescribing of statins, ACEI or ARBs) associated with hypertension status. Analysis was stratified by CKD Stage. Differences between categorical variables were assessed using Pearson’s chi-squared test (${x}^{2}$) (Tables 1 and 2, Table S2, and Table S3). A two-sided p value < 0.05 was considered statistically significant.

Partially adjusted (adjusted for age-group and gender) and fully adjusted (adjusted for age-group, gender, and all other covariates are included in Table 3) logistic regression was conducted to examine the association between systolic blood pressure control in hypertensive CKD patients with demographic factors, selected comorbidities and treatments recommended by the NICE guidelines for cardiovascular risk management in CKD patients (Table 3 and Table S4). A two-sided p value < 0.05 was considered statistically significant.

We excluded anti-hypertensive pharmacotherapy from the logistic regression approach due to ethnicity-guided prescribing, as we had already adjusted for ethnicity. The analysis adjusted for demographic and life-style factors and comorbidities in the final model. All analyses were conducted using STATA 15.

## Results

### Descriptive characteristics of the study population

**Fig. 1:**
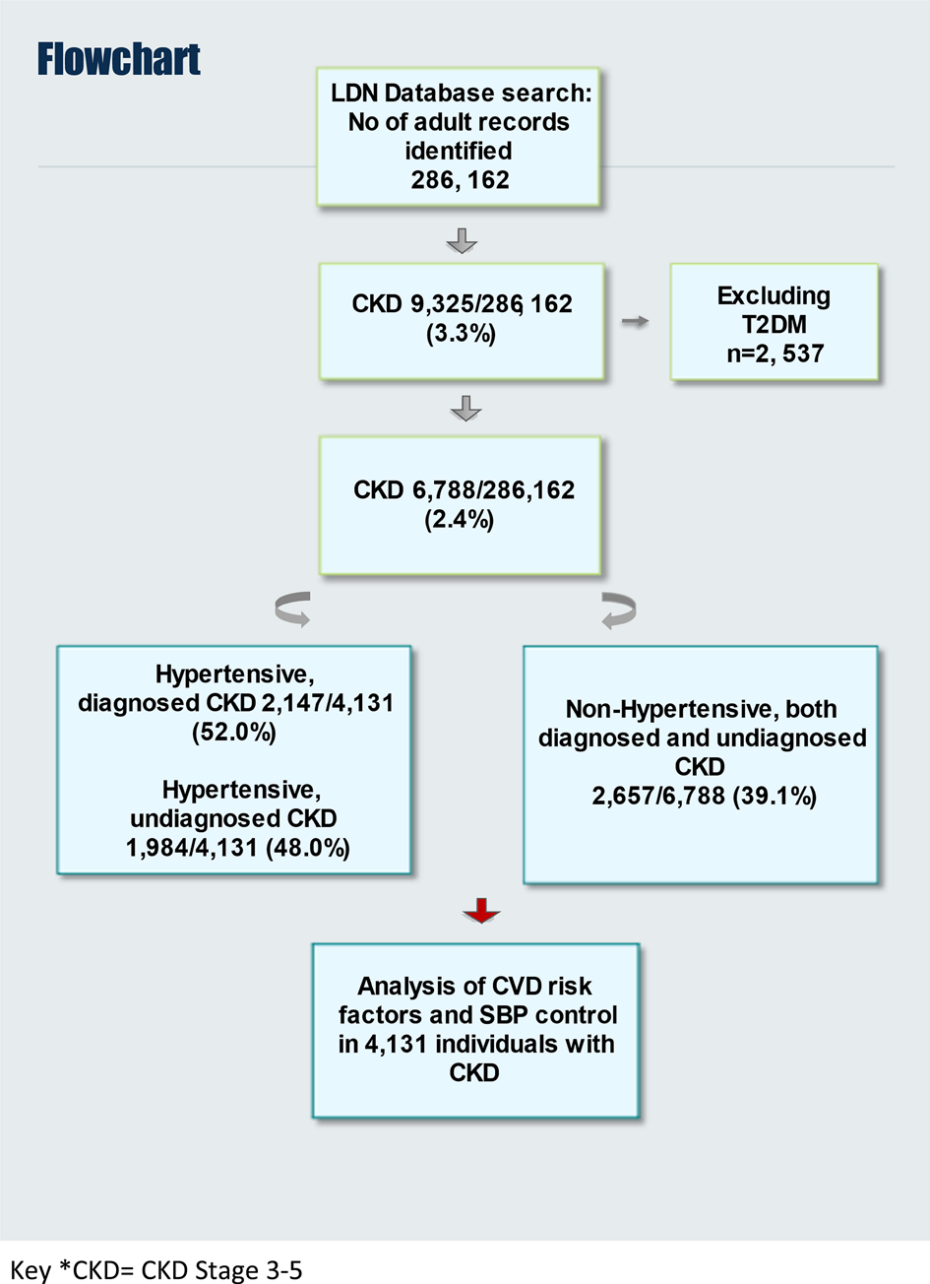
Patient data selection process

Figure 1 shows the flowchart of how patient were selected. The population consisted of 286,162 adults followed in 47/49 GP practices in Lambeth 4131 patients out of 286,162 individuals (1.4%) had CKD (both diagnosed and undiagnosed) and hypertension, nearly half of them (48%, n = 1984) had undiagnosed CKD, as defined by eGFR < 60 ml/min in at least two determinations, without a recorded CKD diagnosis. Figure S1 illustrates the prevalence of CKD in the different age groups in individuals with concurrent hypertension. Figure S2 shows the relationship between age and CKD and hypertension, whose prevalence is rising steeply after 60 years.

### CVD risk factors and comorbidities (Tables 1, 2)

The majority of individuals with CKD were aged > 75 years. Achievement of systolic blood pressure targets was sub-optimal (60.2% vs. 77.0%), both in patients with hypertensive without acknowledged and coded hypertension.

**Table 1:** Prevalence of CVD risk factors and effective risk factor interventions characteristics in patients with CKD by hypertensive status

Variables	CKD and HT	CKD without acknowledged hypertension	*p*-value
	N (Col %)	N (Col %)	
CKD stage ≥ 3	4131 (16.5)	2657 (1.1)	**
Sex			
Female	2388 (57.8)	1556 (58.6)	P > 0.05
Male	1743 (42.2)	1101 (41.4)	
Age (years)			
< 50	346 (8.4)	720 (27.1)	**
50–59	568 (13.8)	642 (24.2)	
60–69	620 (15.0)	480 (18.1)	
70–79	1,127 (27.3)	406 (15.3)	
80–89	1,149 (27.8)	322 (12.1)	
> 90	321 (7.8)	87 (3.3)	
BMI (kg/m^2^)			
Normal or below normal (below 25 kg/m^2^)	1027 (24.9)	778 (31.9)	**
Overweight (25.0–29.9 kg/m^2^)	1,452 (35.2)	796 (30.0)	
Obesity grade I (30.0–34.9 kg/m^2^)	858 (20.8)	412 (15.5)	
Obesity grade II (35.0–39.9 kg/m^2^)	333 (8.1)	147 (5.5)	
Obesity grade III (≥ 40 kg/m^2^)	149 (3.6)	57 (2.1)	
Missing value	312 (7.6)	399 (15.0)	
Index of Multiple Deprivation 2015 (IMD) Quintile (% coded)			
1 (least deprived)	962 (23.3)	623 (23.5)	P > 0.05
2	915 (22.2)	528 (19.1)	
3	697 (16.9)	463 (17.4)	
4	767 (18.6)	524 (19.7)	
5	788 (19.1)	515 (19.4)	
Missing	2 (0.05)	4 (0.15)	
Ethnicity			
European	1,878 (45.5)	1,141 (42.9)	**
Black African	662 (16.0)	421 (15.8)	
Black Caribbean	879 (21.3)	522 (19.7)	
South Asian	174 (4.2)	109 (4.1)	
Chinese	29 (0.7)	9 (0.3)	
Other ethnicity	66 (1.6)	58 (2.2)	
Non-stated	94 (2.3)	69 (2.6)	
Missing	349 (8.5)	328 (12.3)	
History of comorbidities			
Previous stroke	342 (8.2)	61 (2.3)	**
Cancer	37 (0.9)	31 (1.2)	P > 0.05
Coronary Heart Disease, CHD	602 (14.6)	193 (7.3)	**
Serious mental illness, SMI	164 (4.0)	142 (5.3)	*
Heart failure	345 (8.3)	100 (3.7)	**
Mean SBP < 140 mmHg	1,522 (60.2)	472 (77.0)	**
Mean DBP < 90 mmHg	2,278 (90.1)	576 (94.0)	*
BMI (kg/m^2^)			
BMI > 25 kg/m^2^	2,792 (73.1)	1,412 (62.5)	**
Current or ex-smoker	465 (11.3)	464 (17.6)	**
Alcohol > 14 units/week	29 (0.70)	26 (1.0)	P > 0.05
Prescribed treatments			
NSAIDs	991 (24.0)	780 (29.4)	**
COXIBs	16 (0.4)	12 (0.5)	P > 0.05
ACEIs/ARBs	2,945 (71.3)	335 (12.6)	**
Beta-blockers	1,562 (37.8)	319 (12.0)	**
Calcium channel blockers	2,682 (64.9)	167 (6.2)	**
Diuretics	2,191 (53.0)	145 (5.5)	**
Statins	2,266 (54.9)	618 (23.3)	**

CKD includes both diagnosed and undiagnosed CKD Stage 3–5. Patients with diabetes were excluded

*HT* hypertension, *CHD* coronary heart disease, *BP* blood pressure, *BMI* body mass index, *NSAIDs* non-steroidal anti-inflammatory drugs, *COXIBs* COX-2 inhibitors, *ACEIs* angiotensin-converting-enzyme inhibitors, *ARBs* angiotensin receptor blockers, *CCBs* calcium channel blockers, *IMD* index of multiple deprivation

*P < 0.05

**P < 0.001

**Table 2:** Risk factor management in patients with hypertension and CKD (both diagnosed and undiagnosed), according to age

	CKD3 n (%)	CKD 4 n (%)	CKD 5 n (%)
Mean SBP control (< 140 mmHg)			
Age < 75 years	783 (51.4)	39 (49.4)	84 (90.3)
Age ≥ 75 years	739 (48.6)	40 (50.6)	9 (9.7)
	P > 0.05	P > 0.05	P > 0.05
Mean DBP control (< 90 mmHg)			
Age < 75 years	1,070 (47.0)	50 (45.0)	103 (86.6)
Age ≥ 75 years	1,208 (53.0)	61 (55.0)	16 (13.4)
	**	*	P > 0.05
Smoker (current)			
Age < 75 years	301 (64.7)	18 (66.7)	31 (96.9)
Age ≥ 75 years	164 (35.3)	9 (33.3)	1 (3.1)
	**	*	P > 0.05
Statin prescription			
Age < 75 years	986 (43.5)	43 (40.2)	115 (88.5)
Age ≥ 75 years	1,280 (56.5)	64 (59.8)	15 (11.5)
	**	*	P > 0.05
ACEI or ARB prescription			
Age < 75 years	1,456 (49.4)	70 (50.7)	135 (88.8)
Age ≥ 75 years	1,489 (50.6)	68 (49.3)	17 (11.2)
	P > 0.05	P > 0.05	P > 0.05
Calcium channel blocker prescription			
Age < 75 years	1,435 (53.5)	74 (52.1)	134 (86.4)
Age ≥ 75 years	1,247 (46.5)	68 (47.9)	21 (13.6)
	**	P > 0.05	P > 0.05
Overweight and obese (BMI ≥ 25 kg/m^2^)			
Age < 75 years	1,543 (55.3)	54 (50.5)	103 (85.8)
Age ≥ 75 years	1,249 (44.7)	53 (49.5)	17 (14.2)
	**	P > 0.05	P > 0.05

P-values comparing age < 75 years and ≥ 75 years in each category (column totals by age-group), respectively

*HT* hypertension, *CKD* chronic kidney disease, *SBP* systolic blood pressure, *DBP* diastolic blood pressure, *BMI* body mass index, *ACEI* angiotensin-converting-enzyme inhibitor use, *ARB* angiotensin receptor blocker use, *CCB* calcium channel blocker use

*P < 0.05

**P < 0.001

Table S3 shows CKD Stage and measured eGFR according to demographic variables. The prevalence of hypertensive CKD in stage 3 was higher in females than males (16.1% vs 12.9%, p < 0.001), but prevalence of hypertensive CKD in stage 5 was higher in males than females (1.2% vs 0.6%, p < 0.001.)

### BP control

The proportion of patients who achieved the SBP target was similar in pateints younger or older than 75 years (61.3% versus 59.1%). Achievement of the systolic blood pressure target (< 140 mmHg) was lower in CKD 3–5 in patients of Black African ethnicity compared with patients of White ethnicity (55.7% and 60.5%, respectively, p = 0.02), but was not different when compared with Black Caribbean patients (58.8%, p = 0.5) (Table S2).

### Pharmacotherapy

Three hundred nine/4,131 (7.4%) individuals with diagnosed HT and CKD were not taking any of the anti-hypertensive medications considered in our study. Statins were under-prescribed, and this was more marked in hypertensive vs. non hypertensive CKD patients, receiving statins respectively in 54.9% vs 23.3% of the cases (P < 0.001) (Table 1), the gap was especially important for patients under 75 years (47.8 vs 62.0%, p < 0.001).

### Logistic regression

Multivariable logistic regression analyses of factors associated with systolic blood pressure control are shown in Table 3. The multivariabel logistic regression analyses revealed the following significant associations with SBP control: diastolic blood pressure control (adjusted odds ratio 12.81 (95% CI: 8.52–19.26)) and the following comorbidities: heart failure (OR 1.94), coronary heart disease (OR 1.35), serious mental illness (OR 2.06). Conversely, diagnostic CKD coding was associated with improved SBP control. Factors associated with poor systolic blood pressure control were age ≥ 60 years and Black African ethnicity.

**Table 3:** Multivariable regression (MVR) Associations with Mean SBP control in Hypertensive CKD (non-diabetic) adults

	Univariable OR^1^, 95% CI	Multivariable OR^2^, 95% CI
Age (years)		
< 50	Ref.	Ref.
50–59	1.31 (0.96–1.77)	1.03 (0.65–1.62)
60–69	1.25 (0.94–1.68)	0.48 (0.31–0.76)**
70–79	1.14 (0.88–1.50)	0.41 (0.26–0.62)**
80–89	0.96 (0.73–1.26)	0.29 (0.19–0.45) **
> 90	1.05 (0.74–1.51)	0.34 (0.20–0.58)**
Gender (male)	1.06 (0.94–1.21)	0.99 (0.82–1.20)
IMD quintile		
1.00 least deprived	Ref.	Ref.
2.00	0.93 (0.78–1.12)	0.96 (0.73–1.27)
3.00	1.00 (0.82–1.23)	0.93 (0.69–1.25)
4.00	0.99 (0.81–1.20)	0.97 (0.73–1.30)
5.00 most deprived	1.00 (0.82–1.21)	0.93 (0.69–1.23)
Ethnicity		
White	Ref.	Ref.
Black African	0.80 (0.65–0.97)	0.74 (0.55–0.99)*
Black Caribbean	0.82 (0.70–0.96)	1.03 (0.81–1.30)
Asian	1.04 (0.79–1.36)	1.29 (0.81–2.04)
Chinese	0.72 (0.36–1.44)	0.82 (0.29–2.34)
Other	1.78 (1.03–3.07)	1.10 (0.54- 2.25)
Non-stated	1.06 (0.67–1.67)	1.28 (0.70–2.36)
Lifestyle and risk factors		
DBP control	12.45 (9.04–17.15)	12.81 (8.52–19.26)**
Coded CKD3-5	1.11 (0.97–1.26)	1.37 (1.13–1.65)**
BMI > 25 kg/m^2^	0.81 (0.69–0.95)	0.80 (0.65–1.00)*
Current smoker	0.91 (0.74–1.12)	0.77 (0.57–1.04)
Alcohol > 14units/week	0.63 (0.30–1.32)	1.64 (0.52–5.22)
History of comorbidities		
Stroke	1.06 (0.86–1.32)	1.08 (0.78–1.51)
Heart failure	1.53 (1.23–1.89)	1.94 (1.38–2.74)**
CHD	1.33 (1.12–1.58)	1.35 (1.03–1.77)*
SMI	1.64 (1.15–2.32)	2.06 (1.18–3.61)*

CKD includes both diagnosed and undiagnosed CKD Stage 3–5

*SMI* serious mental illness, *CHD* coronary heart disease, *IMD* index of multiple deprivation, *BMI* body mass index, *CKD* chronic kidney disease, *DBP* diastolic blood pressure, *SBP* systolic blood pressure

*P < 0.05

**P < 0.001

^1^Adjusted for age and gender

^2^Adjusted for all baseline covariates in the table

## Discussion

The first important finding in this large cohort of patients followed up in 47 GP practices in Lambeth SE London is the high prevalence of both underdiagnosed CKD (nearly 50%) and underdiagnosed hypertension (23%) in patients with CKD. Furthermore, systolic blood pressure control was below target in a relevant percentage of cases. Multivariable logistic regression revealed that factors associated with improved systolic blood pressure control in CKD included diastolic blood pressure control, and the presence of comorbidities that are probably associated with a more strict patient control; these include serious mental illness, history of cardiovascular co-morbidities, CKD diagnostic coding, and age < 60 years. African ethnicity and obesity were conversely associated with poorer systolic blood pressure control. The finding of a better blood pressure control in patients with serious mental illness is important as this group is at increased risk of cardiovascular mortality [27].

This is a study of a well characterized ethnically and socially diverse population in 47 GP practices in Lambeth SE London, with extensive recording of clinical data, medication, and patients' characteristics. Our large database allowed us to undertake a detailed exploration of risks in individuals with CKD and hypertension, sorting them by ethnicity, and adding important information on CKD prevalence and management in Black African and Black Caribbean sub-groups.

The study has however several limitations and these limitations are mostly shared by observational real life studies; these include diagnostic misclassifications, lack of data on medication adherence and patients' choices, missing data (e.g., proteinuria) and unmeasured confounders. We tried to correct some misclassifications by revising CKD codes, using MDRD ethnicity-adjusted eGFR values. Furthermore, blood pressure readings were based on the mean of the last two recorded measurements after CKD diagnosis, and BP trajectories may provide better longer-term estimates of BP control in future studies. Currently, smoking cessation services are provided by an external provider, and are poorly coded in our database, and so this information was not included in this study.

Although prescriptions were well captured, we did not have estimates of adherence to the prescribed treatments, which may be modulated by ethnicity and CKD status. Lastly, we were also unable to look at longitudinal outcomes, including morbidity and mortality and start of renal replacement therapy.

We found that the overall prevalence of CKD ≥ 3 (both diagnosed and undiagnosed) amongst adults was 3.3%, which is lower than the national average of 4.3% [9]. As the Lambeth population is much younger; data from Lambeth DataNet suggests 45% of the Lambeth population are aged between 18 and 39 years old, differences in population demographics and/or CKD prevalence in different ethnic groups are likely to explain this variation [28]. However, we also noted CKD is underdiagnosed in general practice, similar to national findings in England [9]. Due to the high prevalence of hypertension-related kidney diseases, we would expect most individuals with CKD to have previously diagnosed hypertension. Our study found that in our non-diabetic CKD cohort 4131 individuals out of 6788 cases with CKD (61.9%) had been diagnosed with hypertension. Of 3142 cases with sufficient BP readings, 1994 (63.5%) reached their systolic blood pressure target (Table 1). However, we found that 23% of CKD patients not recorded as with hypertension had mean systolic blood pressure > 140 mmHg, and, importantly, the prevalence of diagnosed heart failure in this group was 3.7%, clinically relevant, even if lower than the prevalence of 8.3% found in CKD patients recorded as hypertensive (P < 0.05).

Obesity and poor blood pressure control are key risk factors for CKD development [22, 23]. However, differences in CKD prevalence by stage and progression cannot be attributed to BP control alone, and disease progression is likely to be multifactorial. Blood pressure remains one of the key determinants of progression, even if not the only one, and our study shows that current BP targets are not being met in a large proportion of the patients. This observation is of clinical importance, considering also that large studies, like SPRINT [29] and ACCORD-BP [30] suggested that even lower BP targets should be adopted [31]. The STOP ACEI trial, which completed recruiting in 2019, will probably identify whether the use of ACEIs and ARBs is beneficial in Stage 4 and 5 CKD, allowing us modulating these treatments in our population [32]. The NICE guidelines recommend an extensive use of statins in CKD for the prevention of cardiovascular diseases and suggest seeking specialist advice before increasing statin doses in patients with eGFR at or lower than 30 mL/min/1.73 m^2^ [14]. The latest KDIGO guideline does not recommend initiation of statins in CKD 5 patients on dialysis (following SHARP and 4-D trials) [33], although this may change in future. Even with these caveats, the use of statins was relatively low in our population, thus suggesting that there may be room for improvement in this regard.

Raising awareness of the importance of risk factor management in primary care includes more rigorous management of systolic blood pressure, together with a series of potentialy beneficial actions on CKD progression, including smoking cessation, which however was not analysed in our study, and correction of overweight and obesity tailoring pharmacotherapy (statins, ACEI/ARB) is also an important tool to prevent CKD development and slow progression. Public health strategies, and education about CKD are important to minimise preventable morbidity and mortality [34]. Subsequently, improved coding linked to eGFR and proteinuria (no longer incentivised in QOF) may offer opportunities for improving data standards, overall supporting better care [35].

## Conclusions

The main result of this study was to find a high prevalence of underdiagnosed CKD and underdiagnosed hypertension in patients with CKD. In CKD patients with hypertension, poorly controlled blood pressure may contribute to CKD progression and increase the risk of heart failure. Our study shows that there is an unmet need to improve CKD detection and management (relative underdiagnosis of CKD Stage 3) in high-risk groups (< 75 years, Black African, Asian, and “Other” ethnic groups). In our study, management of modifiable risk factors was found to be sub-optimal, particularly in younger patients. This applies also to pharmacotherapy (anti-hypertensives, ACEIs/ARBs, statins), which was found to be under prescribed. Improving risk factor management and establishing public health educational strategies should be a priority, as these interventions may help prevent CKD diagnosis or delay disease progression.

## Supplementary Information

Below is the link to the electronic supplementary material.

Supplementary file1 (DOCX 115 KB)

## Abbreviations

ACEI: Angiotensin-converting-enzyme inhibitor drug
ACR: Albumin creatinine ratio
AOR: Adjusted odds ratio
ARB: Angiotensin-receptor blocker
BMI: Body mass index
BP: Blood pressure
CCB: Calcium channel blocker
CCG: Clinical Commissioning Group
CHD: Coronary heart disease
CKD: Chronic kidney disease
CVD: Cardiovascular disease
COXIB: A type of non-steroidal anti-inflammatory drug (NSAID) that directly targets cyclooxygenase-2 COX-2, an enzyme responsible for inflammation and pain
DBP: Diastolic blood pressure
(e)GFR: (Estimated) glomerular filtration rate
EHR: Electronic health record
ESRD: End-stage renal disease
GFR: Glomerular filtration rate
HF: Heart failure
HT: Hypertension
IMD: Index of multiple deprivation
KCL: King’s College London
KDIGO: Kidney Disease: Improving Global Outcomes
LDN: Lambeth DataNet
MDRD: Modification of Diet in Renal Disease
MPH: Master of Public Health
MVR: Multivariable logistic regression
NA-CKD: Normoalbuminuric chronic kidney disease
NCKDA: National CKD audit
NICE: National Institute for Health and Care Excellence
NSAIDS: Non-steroidal anti-inflammatory medication
QOF: Quality Outcome Framework
READ: A coded thesaurus of clinical terms. There are two versions: version 2 (v2) and version 3 (CTV3 or v3)
RRT: Renal replacement therapy
SBP: Systolic blood pressure
SMI: Serious mental illness
WHO: World Health Organization
