# Supplementary material for: Hypertension and cardiovascular risk factor management in a multi-ethnic cohort of adults with CKD: a cross sectional study in general practice
Source: J Nephrol. 2021 Nov 16;35(3):901–10. doi: 10.1007/s40620-021-01149-0 (PMC8995266; doi:10.1007/s40620-021-01149-0)
Supplement: Supplementary file 1 — Supplementary file1 (DOCX 115 KB) [file 40620_2021_1149_MOESM1_ESM.docx]

**Appendix:**

**Table Supplementary 1 (S1): CKD descriptive codes**

| code | description | count | percentage |
| --- | --- | --- | --- |
| 1Z12. | Chronic kidney disease stage 3 | 4676 | 1.634039 |
| 1Z13. | Chronic kidney disease stage 4 | 393 | 0.137335 |
| 1Z14. | Chronic kidney disease stage 5 | 208 | 0.072686 |
| 1Z15. | Chronic kidney disease stage 3A | 29 | 0.010134 |
| 1Z16. | Chronic kidney disease stage 3B | 15 | 0.005242 |
| 1Z1B. | CKD stage 3 with proteinuria | 146 | 0.05102 |
| 1Z1C. | CKD stage 3 without proteinuria | 449 | 0.156904 |
| 1Z1D. | CKD stage 3A with proteinuria | 56 | 0.019569 |
| 1Z1E. | CKD stage 3A without proteinuria | 103 | 0.035994 |
| 1Z1F. | CKD stage 3B with proteinuria | 53 | 0.018521 |
| 1Z1G. | CKD stage 3B without proteinuria | 37 | 0.01293 |
| 1Z1H. | CKD stage 4 with proteinuria | 58 | 0.020268 |
| 1Z1J. | CKD stage 4 without proteinuria | 21 | 0.007339 |
| 1Z1K. | CKD stage 5 with proteinuria | 25 | 0.008736 |
| 1Z1L. | CKD stage 5 without proteinuria | 7 | 0.002446 |
| K053. | CKD3 | 2 | 0.000699 |
| K054. | CKD4 | 1 | 0.000349 |
| K055. | CKD5 | 1 | 0.000349 |
| ckd3plus | All CKD 3 plus Read Codes above | 5819 | 2.033464 |
| qof_ckd | QOF CKD = Read code above plus over 18 at time of diagnosis | 5789 | 2.02298 |

**Table Supplementary 2 (S2): CKD Stage (both diagnosed and undiagnosed) in patients with HT by ethnic group, age, and sex**

|  | eGFR measured  n  (col %) | CKD3  n  (col %) | CKD4  n  (col %) | CKD5  n  (col %) |
| --- | --- | --- | --- | --- |
| Sex |  |  |  |  |
| Female | 12,897  (95.1) | 2,189  (16.1) | 93  (0.7) | 81  (0.6) |
| Male | 10,793  (93.7) | 1,490  (12.9) | 85  (0.7) | 141  (1.2) |
| p-value | ** | ** | 0.62 | ** |
| Age |  |  |  |  |
| < 75 | 18,157  (93.7) | 1,761  (9.1) | 87  (0.5) | 195  (1.0) |
| $\boldsymbol{\geq}$ 75 | 5,533  (97.0) | 1,918 (33.6) | 91  (1.6) | 27  (0.5) |
| p-value | ** | ** | ** | ** |
| Ethnicity |  |  |  |  |
| White | 10,920  (95.4) | 1,695  (14.8) | 86  (0.8) | 67  (0.6) |
|  |  |  |  |  |
| Black African | 4,031  (93.7) | 578 (13.4) | 19  (0.4) | 60  (1.4) |
|  |  |  |  |  |
| Black Caribbean | 4,047  (95.7) | 790 (18.7) | 39  (0.9) | 44  (1.0) |
|  |  |  |  |  |
| South Asian | 1,222  (95.0) | 151  (11.7) | 10  (0.8) | 11  (0.9) |
|  |  |  |  |  |
| Chinese | 225  (94.1) | 25  (10.5) | 1  (0.4) | 2  (0.8) |
|  |  |  |  |  |
| Other ethnicity | 584  (93.4) | 48  (7.7) | 3  (0.5) | 12  (1.9) |
|  |  |  |  |  |
| Non-stated | 483  (92.9) | 84  (16.2) | 6  (1.2) | 2  (0.4) |
|  |  |  |  |  |
| Missing | 2,178  (89.7) | 308 (12.7) | 14  (0.6) | 24  (1.0) |

Abbreviations: eGFR = estimated glomerular filtration rate *based on creatinine*; CKD = chronic kidney disease Patients with diabetes were excluded.

HT defined if on QOF Hypertensive register

* = P<0.05, **=P<0.001

*52 individuals had overlapping CKD codes

**Table Supplementary 3 (S3): Achievement of NICE recommended blood pressure target by ethnicity in individuals with CKD and hypertension**

| Ethnicity | White | Black African | Black Caribbean | South Asian | Chinese | Other | Non-stated | Missing |
| --- | --- | --- | --- | --- | --- | --- | --- | --- |
| CKD SBP control^1^  n  % | 681 60.5 | 226  55.7* | 342  58.8 | 75  69.4 | 11 64.7 | 31  70.5 | 41  65.1 | 115  62.5 |
| CKD DBP control^1^  n  % | 1,058  94.0 | 335  82.5 | 500  85.9 | 102  94.4 | 17  100 | 43  97.7 | 58  92.1 | 165  89.7 |

*Achievement of SBP target (<140mm Hg) was lower in CKD 3-5 in patients of Black African compared with White ethnicity (55.7% and 60.5% respectively, p=0.02), but not when compared with Black Caribbean ethnic groups, 58.8%, p=0.5.

CKD includes both diagnosed and undiagnosed CKD Stage 3-5

^1^For those with at least 2 BP readings

Abbreviations: SBP = systolic blood pressure, DBP = diastolic blood pressure.

SBP control <140 mmHg and DBP control <90 mmHg n= % in each ethnic group with RF control

Patients with diabetes were excluded

**Table Supplementary 4 (S4): Multivariable regression associations with Mean SBP control in CKD (non-diabetic) adults adjusted for comorbidities and lifestyle factors**

| **Mean SBP control**  **(<140mmHg)** | **Odds Ratio**^2^ | **[95% Conf. Interval]** | **Odds Ratio**^3^ | **[95% Conf.** **Interval]** |
| --- | --- | --- | --- | --- |
| **Coded CKD3-5** | 1.37** | 1.14-1.64 | 1.37** | 1.13-1.65 |
| **Age (years)** |  |  |  |  |
| <50 | 0.93 | 0.60-1.44 | 1.03 | 0.65-1.62 |
| 50-59 | 0.47** | 0.31-0.73 | 0.48** | 0.31-0.76 |
| 60-69 | 0.40** | 0.26-0.60 | 0.41** | 0.26-0.62 |
| 70-79 | 0.31** | 0.20-0.48 | 0.29** | 0.19-0.45 |
| 80-89 | 0.34** | 0.20-0.57 | 0.34** | 0.20-0.58 |
| **IMD quintile** |  |  |  |  |
| 1.00 least deprived (ref) | **-** | **-** | - | **-** |
| 2.00 | 0.96 | 0.74-1.25 | 0.96 | 0.73-1.27 |
| 3.00 | 0.94 | 0.71-1.25 | 0.93 | 0.69-1.25 |
| 4.00 | 0.97 | 0.74-1.28 | 0.97 | 0.73-1.30 |
| 5.00 most deprived | 0.96 | 0.73-1.27 | 0.93 | 0.69-1.23 |
| Gender (Male) | 0.98 | 0.81-1.17 | 0.99 | 0.82-1.20 |
| Ethnicity (White ref) | - |  | - | - |
| African | 0.79 | 0.60-1.04 | 0.74* | 0.55-0.99 |
| Afro Caribbean | 0.99 | 0.79-1.24 | 1.03 | 0.81-1.30 |
| Asian | 1.46 | 0.93-2.29 | 1.29 | 0.81-2.04 |
| Chinese | 0.95 | 0.34-2.64 | 0.82 | 0.29-2.34 |
| Other | 1.10 | 0.55-2.19 | 1.10 | 0.54-2.25 |
| Non-stated | 1.25 | 0.71-2.19 | 1.28 | 0.70-2.36 |
| **History of comorbidities** |  |  |  |  |
| Stroke | 1.12 | 0.81-1.54 | 1.08 | 0.78-1.51 |
| Heart failure | 1.90** | 1.37-2.64 | 1.94** | 1.38-2.74 |
| CHD | 1.27 | 0.98-1.64 | 1.35* | 1.03-1.77 |
| SMI | 2.24** | 1.30-3.87 | 2.06* | 1.18-3.61 |
| Lifestyle and risk factors |  |  |  |  |
| Mean DBP control (<90mmHg) | 12.34** | 8.38-18.17 | 12.81** | 8.52-19.26 |
| BMI> 25kg/m2 | - | - | 0.80* | 0.65-1.00 |
| Current smoker | - | - | 0.77 | 0.57-1.04 |
| High alcohol |  |  | 1.64 | 0.52-5.22 |

CKD includes both diagnosed and undiagnosed CKD Stage 3-5

^2^ adjusted for all baseline covariates in the table comorbidities

^3^ adjusted for all baseline covariates in the table comorbidities and lifestyle factors

* = P<0.05, **=P<0.001

**Figure Supplementary 1 (Fig. S1): CKD prevalence in those with and without hypertension, by age-group (all adults ≥18 years)**

**Figure Supplementary 2 (Fig. S2): Prevalence of CKD Stages in patients with hypertensive CKD (all adults ≥18 years)**

CKD includes both diagnosed and undiagnosed CKD Stage 3-5*.

*CKD Stage 5 includes all transplant and dialysis patients
